# Electrospun/3D-Printed Bicomponent Scaffold Co-Loaded with a Prodrug and a Drug with Antibacterial and Immunomodulatory Properties

**DOI:** 10.3390/polym15132854

**Published:** 2023-06-28

**Authors:** Elena Cojocaru, Jana Ghitman, Gratiela Gradisteanu Pircalabioru, Anamaria Zaharia, Horia Iovu, Andrei Sarbu

**Affiliations:** 1 Advanced Polymer Materials Group, University Politehnica of Bucharest, 1-7 Gh. Polizu Street, 011061 Bucharest, Romania; 2 eBio-Hub Research Center, University Politehnica of Bucharest—CAMPUS, 6 Iuliu Maniu Boulevard, 061344 Bucharest, Romania; 3 Research Institute of the University of Bucharest (ICUB), University of Bucharest, 91-95 Splaiul Independentei, 050095 Bucharest, Romania; 4 Academy of Romanian Scientists, 54 Splaiul Independentei, 050094 Bucharest, Romania; 5 Advanced Polymer Materials and Polymer Recycling Group, National Institute for Research & Development in Chemistry and Petrochemistry ICECHIM, 202 Splaiul Independentei, 060021 Bucharest, Romania

**Keywords:** 3D-printing, electrospinning, bicomponent scaffold, immunomodulatory activity, antibacterial activity, wound dressing

## Abstract

This work reports the construction of a bicomponent scaffold co-loaded with both a prodrug and a drug (BiFp@Ht) as an efficient platform for wound dressing, by combining the electrospinning and 3D-printing technologies. The outer component consisted of a chitosan/polyethylene oxide-electrospun membrane loaded with the indomethacin–polyethylene glycol–indomethacin prodrug (Fp) and served as a support for printing the inner component, a gelatin methacryloyl/sodium alginate hydrogel loaded with tetracycline hydrochloride (Ht). The different architectural characteristics of the electrospun and 3D-printed layers were very well highlighted in a morphological analysis performed by Scanning Electron Microscopy (SEM). In vitro release profile studies demonstrated that both Fp and Ht layers were capable to release the loaded therapeutics in a controlled and sustained manner. According to a quantitative in vitro biological assessment, the bicomponent BiFp@Ht scaffold showed a good biocompatibility and no cytotoxic effect on HeLa cell cultures, while the highest proliferation level was noted in the case of HeLa cells seeded onto an Fp nanofibrous membrane. Furthermore, the BiFp@Ht scaffold presented an excellent antimicrobial activity against the *E. coli* and *S. aureus* bacterial strains, along with promising anti-inflammatory and proangiogenic activities, proving its potential to be used for wound dressing.

## 1. Introduction

The skin represents the human body’s largest organ and a vital natural barrier against external factors, fulfilling a protective function [1]. A wound is considered to be the result of skin structure destruction, which can be caused by a variety of harmful internal and external stimuli [2]. Therefore, the reconstruction of the skin tissue integrity remains a widespread clinical problem nowadays, which is addressed by skin tissue engineering, a powerful approach capable of advancing wound healing, with the aim of ensuring the promotion of the human life quality.

Wound healing expresses a complex, interactive, and regenerative process that can be accomplished through the synchronized unfolding of hemostasis, inflammation, proliferation, and re-epithelialization [3]. Wound dressings highlight a versatile and available therapeutic method, used to facilitate and promote the healing process by protection against bacterial invasion and physical abrasion. A wound dressing material may be considered as ideal if it presents several features, e.g., the ability to maintain water balance, good permeability to gases, strong absorption of exudates, capacity to enhance cell proliferation and attachment, good biocompatibility and biodegradability, as well as antimicrobial activity [4]. Instead, conventional wound dressings do not meet all these conditions, due to their drastic adhesion to the skin, minimal fluid absorption, and requirement of repetitive changes, while hydrogel materials [5], electrospun membranes [6], and 3D-printed scaffolds [7] are extensively studied as alternative wound dressing architectures that can influence wound dressing morphology, swelling, and degradation rates, drug loading and release, cellular behavior, and wound secretions absorption.

Electrospinning is a versatile technology that allows the design of nanofibrous materials with primordial features for biomedicine, e.g., high specific surface area, high degree of porosity, versatility for the incorporation of bioactive compounds, and controlled release of loaded biomolecules [8]. In addition, electrospun structures are characterized by advanced biomimetic characteristics and may assure support and optimal conditions for cell attachment and proliferation, which makes them important biomaterials for promoting skin tissue regeneration, with applications in the wound dressing area [9,10].

Three-dimensional (3D) printing represents a personalized and accurate additive manufacturing technique that enables the biofabrication of sophisticated architectures in a layer-by-layer manner, which can fit perfectly with the most complex geometry of any wound [11]. Particularly, 3D-printed and biocompatible hydrogels offer special benefits for wound dressings due to their ability to mimic the native extracellular matrix (ECM) and its porosity and swelling properties, which facilitates the diffusion of water-soluble drugs from the biomaterial and their absorption at the wound site, stimulating skin tissue repairing and promoting their wide use in wound healing [12,13].

The integration of electrospinning and 3D-printing methods represents an exciting strategy for designing dressings as wound healing effective solutions, which brings multiple advantages (high porosity, permeability, and structural similarity of the nanofibrous membrane with the ECM, along with great porosity due to interconnected pores and the ability to maintain a moist medium and swell in order to promote drug diffusion from the 3D-printed hydrogel), not only from the architectural and structural point of view, as this combination may also improve the therapeutic performances of biomaterials, ensuring at the same time the optimal conditions for cell adhesion, proliferation, and differentiation [14]. For instance, Zhang and co-workers [15] developed an asymmetric wound dressing made of a dense polycaprolactone (PCL)/polylactic acid (PLA) outer layer obtained by electrostatic spinning and a porous sodium alginate (SA)/polyvinyl alcohol (PVA)/chitosan quaternary ammonium salt (HACC) inner layer obtained by 3D-printing, which demonstrated similarity to the gradient structure of skin layers and comparable mechanical properties to native human skin. Cao et al. [16] designed a multifunctional wound dressing by 3D-printing [2-(acryloyloxy) ethyl] trimethylammonium chloride (Bio-IL) and gelatin methacryloyl conductive hydrogel strips onto a ROS-responsive polyurethane (PFKU) electrospun membrane surface loaded with doxycycline (DOXH), having electrical conductivity and immunomodulatory properties and applications in diabetic wound treatment in vivo. Then, the same strategy was used to construct a hybrid scaffold consisting of a heparin-conjugated PCL/gelatin electrospun membrane and a PCL/gelatin/nano-hydroxyapatite 3D-printed scaffold capable to promote the repair and regrowth of bone in a rabbit bone defect model [17].

Song et al. synthesized folic acid-functionalized hollow polymeric capsules (FA-HPCs) for drug delivery purposes. In vitro experiments conducted on cancer cells revealed that FA-HPCs showed enhanced cellular uptake and intracellular release of doxorubicin compared to un-functionalized HPCs [18]. The literature reports numerous drug delivery systems, including polypeptide vesicles (PVs) and outer membrane vesicles (OMVs), with antibacterial properties as well as biodegradability and excellent biocompatibility. Moreover, the capacity of PVs and OMVs to respond to particular stimuli, including physical (light, heat), chemical (pH, reducing agents), and biological factors (ATP, RNA), allows an optimal approach for the controlled release of the encapsulated drug [19].

Ganguly and co-workers used the 3D-printing technique to develop magnetic polymeric composite hydrogels for hyperthermia, with shape-morphing behaviors; the entrapment of magnetic nanoparticles into a polymeric matrix led to the remotely controlled pulsatile release of therapeutic charges [20]. The same researcher synthesized polydopamine (PDA)-coated halloysite nanotubes (HNTs), which were subsequently dispersed within a sodium alginate matrix and ionically crosslinked, as potential drug delivery vehicles. The concept of employing nanoscale reservoirs provided by HNTs presents exciting prospects for controlled drug loading, encapsulation, and the release of calcium channel blockers [21].

Another study developed a thermosensitive in situ gelling system containing poloxamer-407 (PM) and a xanthan gum (XG)–guar gum (GG)-based polymer matrix for the sustained release of ophthalmic drugs. The findings from both in vitro and in vivo studies demonstrated that the XG–GG combination in PM significantly enhanced the system’s drug retention capability compared to PM alone, making it a superior alternative to conventional eye drops [22]. Sarkar et al. prepared a polymer film using cellulose nanofibrils (CNFs) as a drug controlled-release carrier in a chitosan matrix for application in transdermal drug delivery systems. Studies showed that CNFs could considerably extend the release time of ketorolac tromethamine (KT) [23].

Chitosan (CS) is recognized as a versatile bio-polysaccharide endowed with intrinsic properties, such as antihemorrhagic, antibacterial, and anti-inflammatory activity, biodegradability, biocompatibility, and muco-adhesion, as well as excellent wound healing ability [24]. Sodium alginate (SA) represents a biocompatible and biodegradable polysaccharide with high liquid-absorbing capability, but due to its minimal mechanical properties and poor cellular adhesion (lack of cell-binding motifs), it is not considered an adequate material for applications in wound dressings [25]. Gelatin methacryloyl (GM) is a collagen derivative with high biomimetic properties, capable to sustain cell attachment, multiplication, migration, and differentiation owing to its RGD (the tripeptide Arg–Gly–Asp) binding motifs [26], while the methacrylic functionalities inserted on the polymer backbone provide the possibility to improve its stability through the photo-crosslinking process.

The present research study aimed to develop a bicomponent scaffold (BiFp@Ht) co-loaded with an anti-inflammatory prodrug (IMC-PEG-IMC) and an antibacterial therapeutic (tetracycline hydrochloride—TCH) as an efficient wound dressing, by combining the electrospinning and 3D-printing technologies. The outer component of the biomaterial consisted of chitosan/polyethylene oxide nanofibrous membrane loaded with the IMC-PEG-IMC prodrug (Fp), while the inner component was formed by a TCH-loaded gelatin methacryloyl/sodium alginate 3D hydrogel (Ht), that was achieved by 3D-printing onto the electrospun membrane surface.

**Figure 1:**
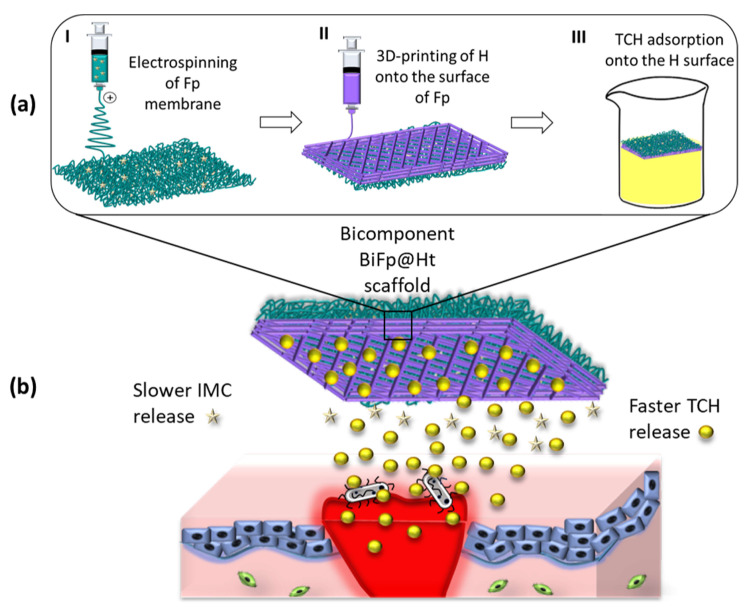
(**a**) Scheme for obtaining the bicomponent scaffold (BiFp@Ht) by the electrospinning and 3D-printing technologies and (**b**) its in vitro application.

To the best of our knowledge, a bicomponent scaffold composed of a printed biopolymeric layer as a 3D hydrogel and an electrospun one as a nanofibrous membrane, both loaded with different therapeutics, used as an effective platform for wound dressing, has not yet been reported in the literature. Likewise, the therapeutic agents were assembled within the polymer matrices in distinct ways in order to facilitate their release depending on the layer that would encounter the wound and due to their different action mechanisms. On the one side, tetracycline hydrochloride (TCH), an effective antibacterial drug used to treat skin infections that locally acts by inhibiting bacterial protein synthesis and further exhibits antioxidant and anti-apoptotic activities [27], was immobilized onto the surface of the 3D-printed hydrogel through physical adsorption. On the other side, indomethacin (IMC), an anti-inflammatory drug, was used as IMC-PEG-IMC prodrug (pIMC) after chemically coupling with PEG, in order to extend its bioavailability and effectiveness, and was incorporated within the CS/PEO polymer blend solution before the electrospinning process (Figure 1). Through this design, we expected to achieve a faster release of the antibacterial drug TCH when the material was in close contact with the wound, thus avoiding infection processes of the injury, a crucial factor in regeneration, and a slower and controlled release of IMC to assure an efficient immunomodulatory activity of the entire system. The potency of the electrospun/3D-printed bicomponent scaffold (BiFp@Ht) co-loaded with an anti-inflammatory prodrug and an antibacterial drug to serve as an efficient platform for wound dressing was further examined. The in vitro release profiles of IMC and TCH in the presence of enzymes were thoroughly studied, and the architectural features of the electrospun and 3D-printed components were highlighted by a SEM morphological analysis. Additionally, the MTT, LDH, and Live/Dead assays were employed in the examination of the cellular response to the BiFp@Ht scaffold in HeLa cell cultures, whereas the system’s anti-inflammatory and proangiogenic activities were evaluated by quantifying the IL-8 and VEGF-A levels using an ELISA test, and its antimicrobial potential was tested against *E. coli* and *S. aureus* bacterial strains.

## 2. Experimental Procedure

### 2.1. Materials

Chitosan (CS) with medium molecular weight (M_w_) and a 75–85% degree of deacetylation, polyethylene oxide (PEO) with M_w_ = 900 kDa, gelatin (Gel) from bovine skin, gel strength ~225 g Bloom, sodium alginate (SA), tetracycline hydrochloride (TCH), indomethacin (IMC, ≥99% TLC), poly(ethylene glycol) diamine (NH_2_-PEG-NH_2_) with M_w_ = 3 kDa, N-(3-dimethylaminopropyl)-N′-ethyl-carbodiimide hydrochloride (EDC, ≥98%), N-hydroxy-succinimide (NHS, 98%), calcium chloride (CaCl_2_, ≥93%), 2-hydroxy-4′-(2-hydroxyethoxy)-2-methyl-propiophenone (water-soluble photo-initiator Irgacure 2959, 98%), triethylamine (TEA, ≥99.5%), methacrylic anhydride (MA, 94%), acetic acid (99.8–100.5%), glutaraldehyde grade I (GA, 50% aqueous solution), deuterium oxide (D_2_O), deuterated dimethyl sulfoxide (DMSO-d_6_), dialysis sacks with an average flat width of 35 mm and MWCO of 3.5 kDa and 12 kDa were acquired from Sigma-Aldrich (Sigma-Aldrich Chemie GmbH, Steinheim, Germany). N, N-dimethylformamide (DMF) for the synthesis of the prodrug was provided by Merck Schuchardt, and ultrapure water was obtained by a Milli-Q Plus system (Millipore, Burlington, MA, USA).

### 2.2. Design of the Electrospun pIMC-Loaded CS/PEO Nanofibrous Membrane (Fp) (as the Outer Component)

The electrospinning solutions were prepared by blending a 3% (*w*/*v*) CS solution and a 3% (*w*/*v*) PEO solution in a 3:7 (*v*/*v*) ratio, according to our previous study [28], to achieve a CS/PEO fibrous membrane (control sample), hereinafter abbreviated as “F”. Subsequently, a 5% (*w*/*v*) IMC-PEG-IMC prodrug (pIMC) (the synthesis protocol and characterization are presented in Supplementary Materials (Figure S1a–c)) was incorporated within the CS/PEO polymeric matrix through magnetic stirring, to obtain the “Fp” nanofibrous membrane. The systems were subjected to the electrospinning process by means of a Climate-Controlled Electrospinning equipment (IME Technologies, Waalre, The Netherlands). The temperature was set to 25 °C, the relative humidity to 40 ± 5%, the applied voltage was in the range of 14–20 kV, and the collecting distance was fixed at 15 cm, while the nanofibers were deposited on a grounded collector with a rotation speed set to 150 rotations per minute (rpm). The electrospun membranes were chemically crosslinked in GA vapors [29], followed by a vigorous washing step with pure water, and structurally characterized by FTIR spectrometry (Supplementary Materials (Figure S1d)).

### 2.3. Building of the 3D-Printed GM/SA Hydrogel Loaded with TCH (Ht) (as the Inner Component)

The printing ink was formulated from a mixture of gelatin methacryloyl (GM) and sodium alginate (SA) solutions (GM/SA) in a 1:1 (*v*/*v*) ratio. GM (the synthesis and structural characterization are presented in Section 2 of the Supplementary Materials) was dissolved in PBS at 37 °C to obtain a 10% (*w*/*v*) GM solution. The pre-crosslinked SA solution was prepared by dissolving 5% (*w*/*v*) SA in a 0.3% (*w*/*v*) CaCl_2_ aqueous solution, at 60 °C with magnetic stirring. Subsequently, 0.5 wt% photo-initiator (Irgacure 2959) by the total solid mass of GM was added to the obtained ink, followed by stirring overnight in dark conditions for complete solubilization. Before printing, the obtained ink system was charged into a 5 mL cartridge provided with a metal needle of 23 G (inner diameter of 0.33 mm, length of 6.35 mm), which was then fitted to the direct dispensing print-head (XYZ moving arm) of the 3D Discovery bioprinter (RegenHU, Villaz-St-Pierre, Fribourg, Switzerland). The 3D hydrogels with a height of 1.65 mm were printed in a controlled layer-by-layer manner on glass plates at room temperature, using optimized pneumatic pressure (120 ± 2 kPa) and printing speed (10 mm/s), by means of BioCAD 1.1 version and BioCAM 1.0 version software packages.

Afterwards, the 3D-printed hydrogels were subjected to a two-step crosslinking: first, GM photopolymerization using an ultraviolet (UV) lamp (365 nm) for 5 min, and second, ionic gelation of SA using a 2% CaCl_2_ solution for 10 min, followed by rinsing several times with ultra-pure water. Subsequently, the TCH loading of the hydrogels was achieved through physical adsorption, by immersing the double-crosslinked hydrogel (H) in a TCH aqueous solution (5 mg/mL), at room temperature for 24 h in dark conditions. The resulted TCH-loaded double-crosslinked hydrogel (Ht) was taken out, washed, and lyophilized; the double-crosslinked hydrogel (H) was used as a control sample. Then, the chemical structure of the H and Ht 3D-printed scaffolds was examined by means of FTIR spectrometry (Supplementary Materials (Figure S2d)).

### 2.4. Construction of the Bicomponent Scaffold (BiFp@Ht)

The assembling of the two components previously described was accomplished by 3D-printing the hydrogel (H) onto the surface of the Fp nanofibrous membrane, under the same technical conditions (Figure 1). Afterward, double-crosslinking of the hydrogel and TCH loading onto the surface of the 3D-printed hydrogel were performed, following the same protocol presented before. Finally, the BiFp@Ht scaffold thus obtained was lyophilized for subsequent analyses.

### 2.5. Characterization Methods

#### 2.5.1. Morphological Investigations

The morphological characteristics of all analyzed samples were examined on a Hitachi TM4000plus II tabletop Scanning Electron Microscope (SEM) (Spectral, Lidingo, Sweden), equipped with a cooling stage and operated at 15 kV. Before the SEM analysis, the samples were covered with an electrically conductive thin film of gold to inhibit “charging”, reducing thermal damage and increasing the emission of secondary electrons.

#### 2.5.2. Wettability Assessment

The wetting properties of the crosslinked and un-crosslinked electrospun nanofibrous membranes were assessed by the Drop Shape Analyzer–DSA100 (Krüss Scientific GmbH, Hamburg, Germany) equipped with a CF03 digital camera. The static contact angle was measured for 20 s at room temperature, after deposition of a water droplet with a volume of 2 µL on the sample surface, using the Advance 1.7.2.1. version software and sessile drop method. The reported results were calculated by means of the Young–Laplace equation and averaged after three measurements.

#### 2.5.3. In Vitro Swelling and Degradation Studies

The in vitro swelling and degradation studies of the materials were performed in PBS solution, mimicking the biological conditions (37 °C and pH 7.4). Three specimens of each sample were lyophilized, weighted (w_0_), and placed in 10 mL of PBS.

The scaffolds were removed from PBS, gently blotted with absorbent paper to eliminate the residual liquid, and weighted in the swelling state (w_s_) at predetermined times (0.5, 1, 2, 3, 4, 5, 6, 8, and 24 h). The maximum swelling degree was considered when the samples’ masses remained constant after two consecutive weightings. The swelling degree (SD) for each sample was quantified according to Equation (1):

(1) $$\mathrm{SD}\;(\%)=\frac{\mathrm{Ws}\;-\;W0}{W0}\;\times \;100$$

The degradation studies were achieved in the presence of the two enzymes (10 U/mL of α-chymotrypsin and 3 U/mL of collagenase). Every 24 h, the scaffolds were removed from PBS, dried by lyophilization, and weighed (w_d_). The degree of degradation (D) was determined according to Equation (2):

(2) $$D\;(\%)=\frac{W0-\;\mathrm{Wd}}{W0}\;\times \;100$$

#### 2.5.4. Drug Loading

The amount of drugs loaded into the scaffolds was quantified by Ultraviolet–Visible Near-Infrared (UV-Vis NIR) spectrophotometer (UV-3600, Shimadzu, Kyoto, Japan). To determine the IMC loading efficiency, a known amount of electrospun scaffold was solubilized in DMSO, and the intensity signal at 360 nm was measured, while for the loading percent of TCH, the drug aqueous solution after extracting the scaffold was analyzed at 280 nm.

#### 2.5.5. In Vitro Release Studies

The in vitro IMC release from both pIMC and Fp was investigated in PBS. Thus, 5 mg each of pIMC and Fp was transferred to dialysis bags (WCO 3.5 kDa) containing PBS, PBS and 10 U/mL α-chymotrypsin, or PBS and 3 U/mL collagenase, which were immersed in the release medium and subjected to magnetic stirring (100 rpm) at 37 °C. At certain intervals, 4 mL of each release medium was extracted and replaced with fresh one, to keep the sink conditions. The in vitro release profile of TCH from Ht was also investigated, following the above-described protocol. The amounts of released IMC and TCH were quantified by UV–Vis spectrometry. The cumulative release of IMC and TCH against the release time was plotted.

#### 2.5.6. Evaluation of the Cellular Response (MTT, LDH, and Live/Dead Assays)

Initially, HeLa human cells (1 × 10^5^ cells/well) were cultured in 24-well plates, in Dulbecco’s Modified Eagle’s medium (DMEM) supplemented with 10% fetal bovine serum and a 1% penicillin–streptomycin solution at 37 °C, in a humid atmosphere with 5% CO_2_ for 24 h. Then, all samples were co-cultured with HeLa cells in the same conditions for 72 h, to allow cell attachment. In vitro cytocompatibility was investigated using the 3-(4,5-dimethylthiazol-2-yl)-2,5-diphenyltetrazolium bromide (MTT) assay (Vybrant^®^ MTT kit from ThermoFisher Scientific, Foster City, CA, USA), after 24 and 72 h of culture. In order to determine the metabolic activity of the cells, the cells were incubated with 1 mg/mL of MTT solution at 37 °C for another 4 h, then the MTT tetrazolium dye was quantified by measuring the absorbance at 550 nm using a Mulsiskan FC spectrophotometer (Thermo Scientific, Waltham, MA, USA). To examine the cytotoxic response, the lactate dehydrogenase (LDH) assay was used, by blending the culture media with the components of a cytotoxicity detection kit (Roche, Rotkreuz, Switzerland), following the producer’s instructions, and incubating the resulting solutions in dark conditions for 20 min, followed by a spectrophotometric analysis at 490 nm. The experiments were carried out in triplicate. The unstimulated cells were used as a control.

Then, a qualitative analysis of biocompatibility was performed by staining the live and dead cells from the scaffolds with fluorescein diacetate (FDA), by means of a Live/Dead kit (ThermoFisher Scientific, Foster City, CA, USA) according to the producer’s protocol. Cell morphology was studied using a confocal microscope (Carl Zeiss LSM 710, Jena, Germany), and the images were processed by means of Zeiss Zen 2010 software.

#### 2.5.7. Immunomodulatory Activity (Enzyme-Linked Immunosorbent Assay—ELISA)

The immunomodulatory profile of all formulations was evaluated by the quantification of pro-inflammatory interleukin (IL)-8 and vascular endothelial growth factor (VEGF-A). HeLa cells were incubated in the presence of the samples, in an atmosphere with 5% CO_2_ and at 37 °C for 24 h. Then, the supernatant of each studied scaffold was collected, and the levels of pro-inflammatory IL-8 and VEGF-A were measured using the ELISA assay (cat. Number BMS277-2 and # KHC0081, Invitrogen, Waltham, MA, USA); the results are expressed as pg/mL. Sample-free cell cultures were used as a control.

#### 2.5.8. Antimicrobial Activity

The antimicrobial activity of all materials was evaluated against the Gram-positive (G+) *Staphylococcus aureus* ATCC 25923 (*S. aureus*) and Gram-negative (G−) *Escherichia coli* ATCC 25922 (*E. coli*) bacterial strains. Bacteria from glycerol stock solutions were streaked on Mueller Hinton agar to attain 24 h cultures for additional analyses. The expansion of monospecific biofilms was assessed at 4 h after exposure to the studied materials. The samples were cut into square pieces of 8 mm, sterilized by UV exposure for 20 min, immersed in 1 mL of a microbial suspension of ~10^7^ colony-forming units (CFU)/mL, and kept in contact with it for 4 h. Subsequently, the microbial suspensions incubated with the samples of interest were vortexed and diluted in ten-fold series. Then, 10 µL of each dilution was covered with nutrient agar in three exemplars, and the viable cells were counted after 24 h at 37 °C, to achieve the CFU/mL for each sample.

#### 2.5.9. Statistical Analysis

The results are expressed as the mean values of three measurements, with their standard deviation (mean ± S.D.). The statistical analysis was conducted using the one-way ANOVA test from GraphPad Prism 8.0.1 (GraphPad Software Inc., San Diego, CA, USA), and the differences were considered significant if *p* < 0.05.

## 3. Results and Discussion

### 3.1. Characterization of the Outer Component

#### 3.1.1. Morphology of the Fp Nanofibrous Membrane

Biomaterials with a nanofibrous architecture are particularly important for the biomedical field owing to their outstanding characteristics, such as their structural resemblance to the ECM, offering optimal conditions for cell adhesion, growth, migration, and differentiation, their bioactivity owing to a great specific surface area, and their porous morphology.

**Figure 2:**
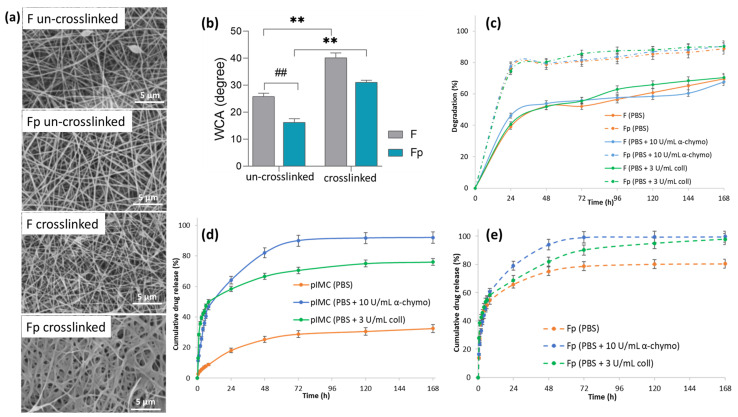
(**a**) SEM micrographs of un-crosslinked and crosslinked nanofibrous membranes, at 7000× magnification; (**b**) water contact angle of the F and Fp un-crosslinked and crosslinked electrospun membranes (## *p* < 0.01; ** *p* < 0.01); (**c**) in vitro degradation of F and Fp in PBS at 37 °C, in the presence of α-chymotrypsin or collagenase for 168 h; in vitro release profile of IMC from (**d**) pIMC, and (**e**) Fp in PBS at 37 °C, in the presence of α-chymotrypsin or collagenase within 168 h.

The morphology of the F and Fp un-crosslinked and crosslinked electrospun membranes is highlighted in Figure 2a. In the case of the un-crosslinked Fp, the addition of pIMC led to continuous, uniform, and beads-free nanofibers, as compared to the un-crosslinked F that showed nanofibers with some beads. The crosslinking step was accomplished by the formation of new covalent bonds between the polymer chains and the GA functional groups and did not significantly change the morphology of the F sample but led to a denser structure in the case of the Fp membrane.

#### 3.1.2. Wettability and In Vitro Degradation of the F and Fp Nanofibrous Membranes

The literature data revealed that scaffolds with enhanced hydrophilic properties are ideal for damaged epidermal tissue repairing, because they allow the absorption of wound exudate and maintain a proper moist environment during the wound healing process [30].

Therefore, static water contact angle measurements for the F and Fp un-crosslinked and crosslinked nanofibrous membranes were performed (Figure 2b) to investigate the influence of the crosslinking agent (GA) and pIMC on the wettability of the membranes’ surface. It was obvious that the crosslinked membranes’ surface was more hydrophobic than that of the un-crosslinked ones, due to the covalent imine (C=N) bonds that formed between the -NH_2_ groups of CS and the aldehyde groups of GA [31]. The contact angle values of the un-crosslinked F and Fp membranes were 25.8° ± 1.29 and 16.3° ± 1.33, respectively, while in the case of the crosslinked membranes, the values of the contact angle were two-fold higher (40.2° ± 1.77 and 30.9° ± 1.02, respectively). Additionally, it was observed that the pIMC-containing membranes, both un-crosslinked and crosslinked, presented a higher degree of hydrophilicity (the contact angle was smaller by approximately 10°) than their standard counterparts, which could be attributed to the hydrophilic character of the loaded prodrug (IMC-PEG-IMC). Overall, the surface of the analyzed nanofibrous membranes displayed a sufficient hydrophilic character to promote cell viability and wound healing through tissue regeneration.

Further, the in vitro enzymatic degradation of the F and Fp nanofibrous membranes was analyzed in PBS medium at 37 °C with and without enzymes (collagenase or α-chymotrypsin), (Figure 2c). It was observed that both F and Fp showed approximately similar mass loss percentages, regardless of the enzymes’ presence, over a period of 168 h. Thus, F presented a degradation degree of 70% in PBS, 68% in PBS containing α-chymotrypsin, and 70% in PBS containing collagenase, whereas Fp degraded in a proportion of 89% in PBS, 91% in PBS containing α-chymotrypsin, and 90% in PBS containing collagenase. Instead, it was observed that Fp showed a higher degradation than F, in all environments studied. According to the water contact angle measurements, being more hydrophilic than the F sample, Fp could absorb a larger water amount and thus degraded faster.

#### 3.1.3. In Vitro Release Study of Fp

The in vitro IMC release profiles of pIMC and Fp in the presence or absence of enzymes are shown in Figure 2d,e. According to the results (Figure 2d and Table 1), pIMC presented an extremely slow release of IMC in PBS, which was only about 9% within 8 h and increased to 32.8% at the end of the experiment (after 168 h of incubation). This low release rate of IMC molecules is assumed to be associated with the much slower hydrolytic cleavage of the stable amide bond formed within the pIMC structure [32]. Instead, the use of proteolytic enzymes (collagenase or α-chymotrypsin), which have the role of hydrolyzing amide linkages [33] formed between the -COOH groups of IMC and the -NH_2_ groups of NH_2_-PEG-NH_2_, led to an accelerated IMC release. The cumulative release profile of IMC from pIMC under enzymatic degradation showed the classical burst release within the first 8 h and then a sustained and controlled release up to 168 h. At the end of the experiment, in the presence of α-chymotrypsin, the amount of IMC released was higher (92%) as compared to the cumulative amount of IMC released in the medium with collagenase (76%).

The in vitro IMC release from the Fp nanofibrous membrane (Figure 2e and Table 1) appeared also to be described by a biphasic model, with a burst release followed by a controlled and sustained one, regardless of the absence or presence of enzymes. The IMC rapid release from Fp (approximately 55%) within the first 8 h, even in the absence of enzymes, could be assigned to the hydrophilicity of the nanofibrous membrane, according to the water contact angle results. Similar observations were reported by Quan, who found that the incorporation of prodrugs within nanofibrous structures led to a faster release of the drug [34]. After 168 h, the amount of IMC released from Fp reached approximately 80%, indicating a controlled and sustained release of IMC through the scaffold, thereby increasing its bioavailability and therapeutic effect [35]. No major differences were observed between the cumulative released amounts of IMC from Fp in the presence of α-chymotrypsin (99.7%) or collagenase (97.9%) until the end of the tests.

### 3.2. Characterization of the Inner Component

#### 3.2.1. Morphology of the 3D-Printed Hydrogels

**Figure 3:**
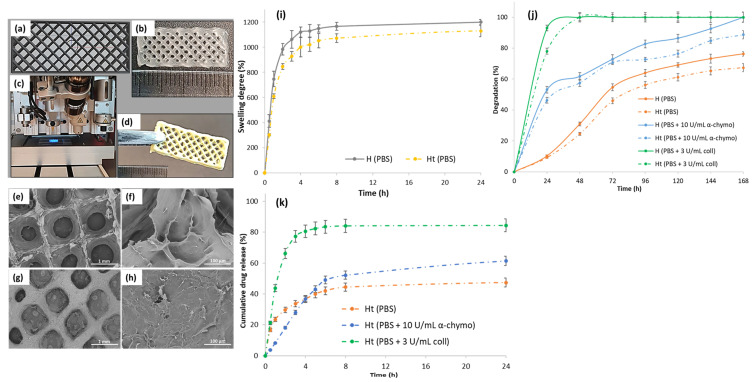
(**a**) Software 3D-printed pattern; (**b**) digital photograph of the 3D-printed hydrogel with dimensions of 20 mm × 8 mm, in 5 layers; (**c**) photo-crosslinking step of the 3D-printed hydrogel using a UV lamp at 365 nm; (**d**) digital photograph of the lyophilized TCH-loaded scaffold; SEM micrographs of the lyophilized (**e**,**f**) H and (**g**,**h**) Ht scaffolds; (**i**) in vitro swelling behavior of the H and Ht 3D-printed scaffolds analyzed in PBS at 37 °C; (**j**) in vitro degradation of H and Ht in PBS, at 37 °C, in the presence of α-chymotrypsin or collagenase for 168 h; (**k**) in vitro TCH release profile from the Ht hydrogel in PBS, at 37 °C, in the presence of α-chymotrypsin or collagenase for 24 h.

In Figure 3a–d, the pattern design and 3D-printed hydrogel along with the lyophilized scaffold loaded with TCH are illustrated. The features of the macro- and micro-internal architecture of the materials are highlighted in the SEM micrographs in Figure 3e–h. It is known that the morphological characteristics of hydrogels influence cellular development and may define their potential application in the biomedical field. Cell viability and proliferation are dependent on the gels’ pore size, and micropores impair the cell nucleus, thus affecting mitotic division in the cell cycle [36]. The 3D-printed H scaffold presented a more porous and rarefied microstructure due to larger pore sizes, compared to the Ht scaffold, which was characterized by a denser and more compact porous microarchitecture. The TCH molecules adsorbed on the Ht scaffold surface were also highlighted in the SEM images.

#### 3.2.2. In Vitro Swelling and Degradation of the 3D-Printed Hydrogels

In vitro swelling and degradation are essential properties of hydrogels, especially for those intended for wound dressing applications, because they provide valuable indications regarding the hydrophilicity of the material surface, aid to promote cell adhesion and cell–cell interactions, and simplify the transfer of nutrients into a wound, as well as can promote the release of loaded active biomolecules [37,38].

The swelling behavior of the H and Ht 3D-printed hydrogels was monitored in PBS, at 37 °C for 24 h (Figure 3i). The samples absorbed a considerable amount of water and swelled in aqueous media, owing to their hydrophilic character. It was observed that both hydrogels exceeded the maximum swelling rate of 1000% of their initial weight, but a slight difference was visualized: H reached a maximum swelling degree of 1200%, whereas Ht reached a swelling degree of approximately 1130%. Studies revealed a more porous microstructure, a higher swelling degree [39], and, according to the SEM results, H presented a more porous microarchitecture than Ht. It is assumed that the lower swelling tendency of Ht could be due to the TCH molecules adsorbed onto the hydrogel surface, which impede the water molecules diffusion into the hydrogel microstructure.

In order to ensure an efficient therapy, it is essential that the drug-loaded scaffolds used as antibacterial wound dressings degrade, allowing the therapeutic agent to be released directly to the injured site and prevent wound infection from an early stage. To this end, the in vitro enzymatic degradation of the H and Ht 3D-printed hydrogels was investigated by incubating the samples in PBS containing collagenase or α-chymotrypsin at 37 °C, and the degradation profiles are displayed in Figure 3j. The obtained results showed that the enzymes’ presence in PBS led to the acceleration of the degradation of all samples as compared to that achieved with the enzyme-free medium. The hydrogels were almost completely degraded after only 24 h of incubation in the presence of collagenase, due to the gelatin included in the hydrogels’ composition, which being derived from collagen, is characterized by a high susceptibility to collagenase digestion/degradation, in accordance with previous studies [40]. In addition, the degradation rate of the Ht hydrogels showed a decreasing trend as compared to that of the H hydrogels, probably because of a lower porosity and, correspondingly, a lower swelling rate. Thus, transposing the previous observations into values, H underwent a 76% degradation in PBS alone and a 100% degradation in PBS containing chymotrypsin and in PBS containing collagenase, whereas the degradation profiles of Ht indicated a mass loss of 67% in PBS alone, 89% in PBS containing chymotrypsin, and 100% in PBS containing collagenase, at the end of the experiment (168 h).

#### 3.2.3. In Vitro TCH Release Studies from the Ht 3D-Printed Hydrogel

In vitro TCH release studies were performed in PBS medium at 37 °C with or without collagenase or α-chymotrypsin (maintaining the same conditions as for the IMC release investigations) for 24 h, and the results are presented in Figure 3k. TCH was gradually released from H in PBS alone, in a ratio of approximately 50% within 24 h, presumably due to the non-covalent interactions that occurred between TCH functionalities (-OH) and those in the polymer matrix constituted by GM (-NH_2_, -OH) and SA (-OH, -COOH), which could obstruct the TCH release. The presence of enzymes (α-chymotrypsin or collagenase) considerably increased the amount of TCH released in the medium. After 24 h, the cumulative TCH release from Ht was about 62% in PBS containing α-chymotrypsin and 85% in PBS containing collagenase; the faster and higher amount of TCH released in the presence of collagenase could be also associated with the higher degradation rate of the hydrogel matrix, in accordance with the degradation experiments.

### 3.3. Characterization of the Electrospun/3D-Printed Bicomponent Scaffold

#### 3.3.1. Morphology of the Bicomponent Scaffold (BiFp@Ht)

**Figure 4:**
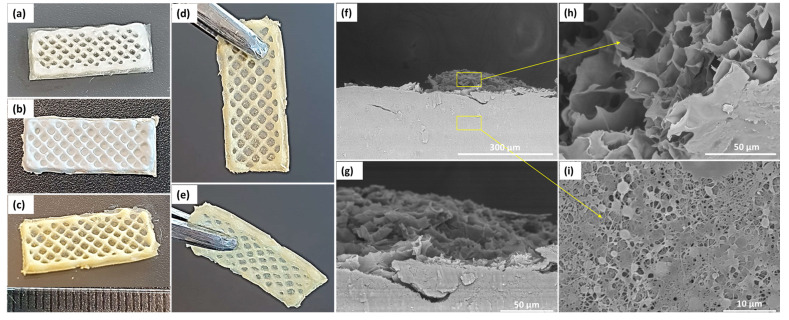
General macro- and microscopic architectural features of the bicomponent BiFp@Ht scaffold. Representative digital photographs of (**a**) TCH-free hydrogel; (**b**) TCH-free lyophilized scaffold; (**c**–**e**) TCH-loaded lyophilized scaffold; SEM micrographs of (**f**), (**g**) lyophilized bicomponent scaffold in cross section at different magnifications, (**h**) 3D-printed hydrogel, and (**i**) electrospun membrane.

The structure of the bicomponent BiFp@Ht scaffold was accomplished by two innovative designing techniques, electrospinning and 3D-printing. The outer component was fabricated by the electrospinning method, obtaining a nanofibrous membrane loaded with pIMC (Fp), on which the Ht inner component was 3D-printed. The macro-morphology of BiFp@Ht is presented in Figure 4a–e, while the cross section of the bicomponent BiFp@Ht scaffold along with the micro-morphology of the two structures are presented in Figure 4f–i. In the recorded SEM images, the nanofibrous continuous architectures of the outer electrospun component and the highly porous structure with interconnected pores in the case of the inner 3D-printed hydrogel were very well highlighted.

#### 3.3.2. Cellular Response Evaluation (MTT, LDH, Live/Dead Assays)

**Figure 5:**
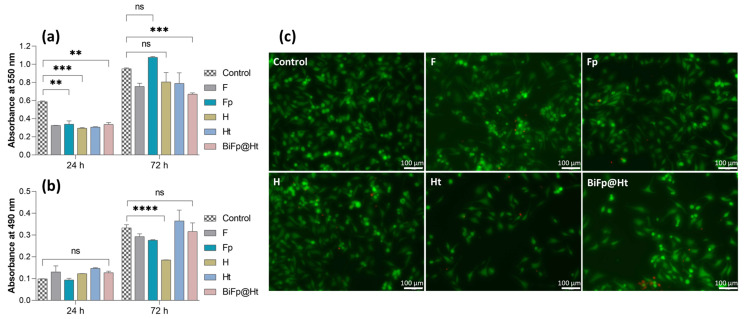
Biocompatibility evaluation of the formulations: (**a**) MTT assay, viability and proliferation potential of HeLa cells cultivated onto the surface of the scaffolds for 24 h and 72 h (ns *p* < 0.5, ** *p* < 0.01, *** *p* < 0.001); (**b**) LDH assay, cytotoxic response of HeLa cells incubated in the presence of the scaffolds for 24 h and 72 h (ns *p* > 0.5, **** *p* < 0.0001); (**c**) qualitative biocompatibility analysis of the samples using confocal microscopy, displaying live (green) and dead (red) cells after 24 h. Scale bar is 100 μm.

A quantitative evaluation of the in vitro cellular response for all formulations was performed on the HeLa cell line, after 24 h and 72 h of incubation with the hydrogels, using the MTT cytocompatibility and LDH cytotoxicity assays, as well as on a standard cell culture as a control (Figure 5).

The MTT assay results (Figure 5a) indicated that after 24 h of culture, the cytocompatibility level of all analyzed structures was lower than that of the control. However, F, H, and Ht presented similar levels of cell adhesion, whereas Fp and BiFp@Ht were distinguished by a slight increase in the cell survival degree. Both the control and all samples showed a significant increase in cell viability and even a good cell proliferation potential after 72 h of contact with the culture medium, suggesting a great degree of cytocompatibility. The highest level of cell growth was reached by Fp that improved the cell viability value by three times in 72 h. This can be attributed to the nanofibrous architecture of the F membrane containing pIMC, which led to an increase in the material hydrophilicity (an indispensable property for cell adhesion) as well as to a slow and gradual release of IMC, without affecting the cells, as reported by previous studies [32].

The cytotoxicity of the samples on HeLa cells was investigated using the LDH assay by measuring the LDH enzyme level released in the culture media (Figure 5b). According to the obtained results, no significant cytotoxicity was registered as compared to the control after 24 h, regardless of the investigated sample. Fp displayed the lowest cytotoxic response, whereas the highest level of LDH was reached by Ht, after 72 h. The presence of the antibacterial compound TCH, adsorbed onto the hydrogel surface, could increase the number of dead cells. Still, considering the extremely low level of cytotoxicity compared to the degree of cell viability, the collected data can sustain the idea that the investigated materials have the ability to promote cell adhesion and proliferation, and could facilitate the healing of a damaged tissue and the reconstruction of new tissue.

A qualitative evaluation of the in vitro cell viability on the materials was accomplished by confocal microscopy (Figure 5c), assessing the Live/Dead staining by visualizing the distribution of the cells within the internal structure of the scaffolds and the ratio of live (green) to dead (red) cells, after 24 h of incubation. The results of the Live/Dead assay are in agreement with the conclusions of the MTT and LDH tests, highlighting the significantly higher number of viable cells as compared to the number of dead ones in all the evaluated samples. The highest proportion of living cells was found on Fp and H, presenting a uniform distribution over the entire surface of the materials. In contrast, Ht displayed a decrease in the live cell number, with live cells unequally arranged, suggesting that this material slightly disturbed cell viability and growth.

#### 3.3.3. In Vitro Investigation of the Immunomodulatory and Antimicrobial Activities of the Materials

**Figure 6:**
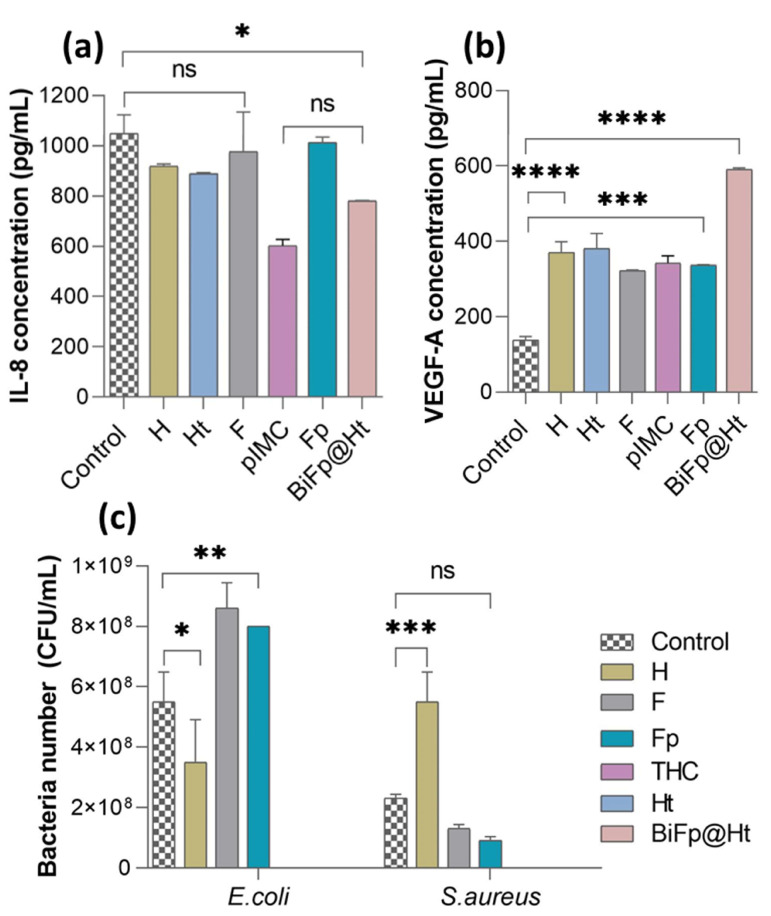
Immunomodulatory properties of the biomaterials assessed by quantifying the level of (**a**) IL-8 (ns *p* > 0.1, * *p* < 0.05) and (**b**) VEGF-A (*** *p* < 0.0005, **** *p* < 0.0001), after 24 h of incubation with HeLa cells; (**c**) evaluation of the antimicrobial activity of the formulations against *E. coli* (* *p* < 0.05, ** *p* < 0.005) and *S. aureus* (ns *p* < 0.5, *** *p* < 0.0005).

The immunomodulatory profiles of the investigated systems were determined by quantifying the protein expression levels of pro-inflammatory IL-8 (pg/mL) and VEGF-A (pg/mL) released into the supernatant medium by HeLa cells after 24 h of incubation with the samples, by ELISA assays, using unstimulated cells asa control. The results of the immunomodulatory activity are summarized in Figure 6a,b.

It is recognized that the IL-8 cytokine is secreted by macrophages as a response to skin injury and participates in the inflammation phase of wound healing [41]. IL-8 also plays a central role in regulating the functions of immune cells from the epithelialization stage, acting by assembling resident stem cells and stimulating cell proliferation and differentiation [42]. It was observed that after 24 h of HeLa cells incubation in the presence of the materials, the amounts of IL-8 secreted were below those measured for the control sample (1050 pg/mL). However, the fact that HeLa cells kept in contact with Ht released a lower amount of IL-8 (886 pg/mL) compared to cells in contact with H (925 pg/mL) is consistent with previous studies, which claimed that TCH exerts an anti-inflammatory action through reducing the secretion of pro-inflammatory markers [43]. pIMC alone exerted the lowest stimulation effect on pro-inflammatory cytokine secretion (584 pg/mL), owing to its intrinsic anti-inflammatory properties, in accordance with the results reported in Lin’s study [32]. The bicomponent BiFp@Ht scaffold induced a fairly low concentration of IL-8 (781 pg/mL) compared to the other samples, which can be attributed to the presence of both pIMC and TCH and may suggest that the BiFp@Ht can control the inflammatory response, promoting the repair the injured tissue.

During wound healing, epithelial cells, fibroblasts, macrophages, and platelets secrete VEGF, which acts in a paracrine manner on endothelial cells, inducing and supporting angiogenesis. In line with this, the level of the proangiogenic factor (VEGF-A) released in the presence of the previously exposed samples was determined to assess their capacity to promote the development of new blood vessels in the wound healing process, which is supported through multiple mechanisms, including collagen-based ECM deposition, angiogenesis, and vasculogenesis, as well as epithelization [44].

In Figure 6b, we show that HeLa cells were stimulated when in contact with all the analyzed samples, releasing significantly higher amounts of VEGF-A compared to the control cells (138 pg/mL). The bicomponent BiFp@Ht scaffold co-loaded with an anti-inflammatory prodrug and an antibiotic led to the highest VEGF level (591 pg/mL), indicative of a positive effect on wound healing. The chemical composition of the BiFp@Ht scaffold can promote a pro-angiogenic effect: gelatin intensely stimulates blood vessel formation, e.g., angiogenesis [45], alginate absorbs large amounts of exudate and maintains a moist wound environment [46], while indomethacin inhibits inflammation, and tetracycline inhibits wound infection. Then, the Ht and H scaffolds were associated with similar values of proangiogenic factor (381 pg/mL and 371 pg/mL, respectively), followed by the F formulation, with the lowest VEGF-A concentration (322 pg/mL). Overall, the obtained results demonstrated that the bicomponent BiFp@Ht scaffold harbors good immunomodulatory properties, including proangiogenic activity.

Further, the antimicrobial activity of the biomaterials was investigated to evaluate their capacity to inhibit the adherence and viability of G− *E. coli* and G+ *S. aureus* bacteria on the scaffolds’ surface. As illustrated in Figure 6c, the antimicrobial activity of the analyzed scaffolds was impacted by both the materials’ composition and the bacterial strain type. The structural differences between the two types of bacteria consist of the following aspects: G− bacteria possess a thin cell wall made of peptidoglycan, enveloped by an outer membrane that contains lipopolysaccharide, phospholipids, and proteins, giving the cell surface negative charges; G+ bacteria do not have an outer membrane, but their cell walls are highly thick and abundant in peptidoglycans, glycolipids, and teichoic acids, which provide the cell surface with positive charges [47].

It was observed that the *E. coli* (G−) bacterial biofilm was less developed on the surface of the H material, compared to that of *S. aureus* (G+), as the latter bacteria adhered to the hydrogel in a larger number. The good antimicrobial activity registered against G− bacteria may be due to the electrostatic repulsion phenomenon occurring between the negative charges of SA and GM in the H structure and the negatively charged *E. coli* bacterial cell surface. On the other hand, the electrostatic attraction between the positive charges of the NH_3_^+^ groups of CS in the F and Fp membranes and the negative charges on the bacterial cell surface may explain the decreased antimicrobial activity of the F and Fp materials against *E. coli* [48]. On the contrary, these samples (F and Fp) exhibited a favorable antimicrobial activity against the positively charged *S. aureus* bacteria. Last but not least, it was observed that the TCH-containing materials displayed good antimicrobial activity, due to the presence of the TCH antibacterial drug [49,50,51,52,53,54,55,56,57,58,59,60], which led to the total inhibition of bacterial viability. Consequently, the Ht and BiFp@Ht bicomponent scaffolds demonstrated an efficient antibacterial activity towards the tested bacteria, regardless of their type.

## 4. Conclusions

In summary, this research work was focused on the design of an electrospun/3D-printed bicomponent scaffold co-loaded with two distinct therapeutic agents, with potential applications in wound dressings. The Fp electrospun membrane and Ht 3D-printed hydrogel constituted, respectively, the outer (in contact with the external environment) and the inner (in contact with the wound) components of the bicomponent biomaterial. The morphology of both components played an important role in the construction of an efficient scaffold used as wound dressing, owing to the high porosity, with interconnected pores, of the inner 3D-printed hydrogel and the large specific surface area of the outer nanofibrous membrane, which led to a structural similarity of the bicomponent scaffold to the ECM, ensuring at the same time optimal conditions for cell adhesion, proliferation, and differentiation. The preservation of electrospun and 3D-printed architectural features of the bicomponent BiFp@Ht scaffold was confirmed by SEM micrographs that highlighted very well the continuous nanofibrous structure of the electrospun component and the highly porous morphology of 3D-printed hydrogel.

The studies of the in vitro drugs release profiles demonstrated that both Ht and Fp components were capable to release the loaded therapeutics (TCH and IMC) in a controlled and sustained manner. On the one hand, the loading of TCH onto the 3D-printed hydrogel led to a faster release of the antibacterial drug when the material was in close contact with the wound, thus avoiding wound infection. On the other hand, the incorporation of IMC as a prodrug (pIMC) within the F nanofibrous membrane led to a controlled and sustained release profile of IMC, increasing its bioavailability and therapeutic effectiveness.

According to the quantitative in vitro biological evaluation (MTT assay), the bicomponent BiFp@Ht scaffold presented a good cytocompatibility and no cytotoxic potential in HeLa cell cultures; the highest cell viability and proliferation level was noted in the presence of the Fp nanofibrous membrane, and the highest level of LDH was observed in the case of the Ht hydrogel after 72 h of incubation, probably due to the increased concentration of the TCH antibacterial drug, which was responsible for killing the cells. In addition, the Live/Dead qualitative assay supported the results achieved by the MTT and LDH tests. Furthermore, the bicomponent BiFp@Ht scaffold exhibited promising anti-inflammatory and proangiogenic activities and an excellent antimicrobial activity against *E. coli* and *S. aureus* bacterial strains. The results of the cellular response assessment proved the potential of the BiFp@Ht scaffold to promote HeLa cells’ adhesion and proliferation, as well as to create new blood vessels to sustain the regeneration of injured skin tissue.

The perspectives of this research study foresee the evaluation of the mechanical performances and water vapor transmission rate, as well as of the in vitro wound healing properties of the bicomponent BiFp@Ht scaffold to confirm its potential in wound dressing applications.

## Figures and Tables

**Table 1:** Summary comparative table regarding the IMC release rates from pIMC and Fp up to 168 h.

Time (h)	% IMC Released from pIMC			% IMC Released from Fp		
	PBS	PBS + α-Chymo	PBS + Coll	PBS	PBS + α-Chymo	PBS + Coll
8	8.8	46.6	50.0	54.6	60.6	58.3
24	18.2	64.1	58.3	65.9	79.2	68.7
48	25.2	82.0	66.6	75.2	94.1	81.9
72	28.7	90.0	70.6	78.8	99.2	90.2
168	32.8	92.0	76.0	80.5	99.7	97.9

## Data Availability

The data presented in this study are available upon request from the corresponding author.

## Supplementary Materials

The following supporting information can be downloaded at: https://www.mdpi.com/article/10.3390/polym15132854/s1, Figure S1: (a) Preparation of pIMC by the EDC/NHS coupling carbodiimide system; structural characterization of pIMC: (b) ^1^H-NMR spectra of IMC, pIMC, NH_2_-PEG-NH_2_, and (c) ATR-FTIR spectra of IMC, pIMC, NH_2_-PEG-NH_2_; (d) ATR-FTIR spectra of pIMC, F, Fp; Figure S2: (a) Preparation of GM. (Abbreviations: Gel—gelatin; MA—methacrylic anhydride; GM—methacryloyl-modified gelatin; MAc—methacrylic acid). Structural characterization of GM: (b) ^1^H-NMR spectra of Gel and GM and (c) ATR-FTIR spectra of Gel and GM; (d) ATR-FTIR spectra of GM, SA, H, TCH, Ht.
