# Supplementary material for: Electrospun/3D-Printed Bicomponent Scaffold Co-Loaded with a Prodrug and a Drug with Antibacterial and Immunomodulatory Properties
Source: Polymers (Basel). 2023 Jun 28;15(13):2854. doi: 10.3390/polym15132854 (PMC10346292; doi:10.3390/polym15132854)
Supplement: Supplementary file 1 [file polymers-15-02854-s001.zip › polymers-2415282-supplementary.pdf]

polymers

MDPI

Supplementary Materials

Electrospun/3D-Printed Bicomponent Scaffold Co-Loaded with a Prodrug and a Drug with Antibacterial and Immunomodulatory Properties

Elena Cojocaru [1], Jana Ghitman [1,2,*], Gratiela Gradisteanu Pircalabioru [2,3,4], Anamaria Zaharia [5], Horia Iovu [1,2,4] and Andrei Sarbu [5]

1 Advanced Polymer Materials Group, University Politehnica of Bucharest, 1-7 Gh. Polizu Street, 011061 Bucharest, Romania; elena.cojocaru3105@upb.ro (E.C.); horia.iovu@upb.ro (H.I.)
2 eBio-hub Research Center, University Politehnica of Bucharest - CAMPUS, 6 Iuliu Maniu Boulevard, 061344, Bucharest, Romania;
3 Research Institute of the University of Bucharest (ICUB), University of Bucharest, 91-95 Splaiul Independentei, 050095 Bucharest, Romania; gratiela.gradisteanu@icub.unibuc.ro (G.G.P.)
4 Academy of Romanian Scientists, 54 Splaiul Independentei, 050094 Bucharest, Romania;
5 National Institute for Research & Development in Chemistry and Petrochemistry ICECHIM, Advanced Polymer Materials and Polymer Recycling Group, 202 Splaiul Independentei, 060021 Bucharest, Romania; anamaria.zaharia@icechim.ro (A.Z.); andrei.sarbu@icechim.ro (A.S.)
* Correspondence: jana.ghitman@upb.ro

1. *Synthesis and characterization of IMC-PEG-IMC prodrug (pIMC)*

The synthesis of IMC-PEG-IMC prodrug was performed through EDC/NHS coupling carbodiimide system, as shown in **Figure S1 a**. Briefly, 24 mg IMC (67 µmol), 25.7 mg EDC (134 µmol) and 15.4 mg NHS (134 µmol) were solubilized in a volume of 3 mL DMF under vigorous stirring after adding each component, at room temperature for 2 h. Simultaneously, 84 mg NH_2-PEG-NH_2 (28 µmol) was dissolved in 3 mL DMF containing TEA under continuous stirring. After 2 h, the activated IMC-containing blend was added to the NH_2-PEG-NH_2 solution, and the reaction was performed in the dark conditions at room temperature for 24 h. The mixture was subjected to dialysis in Milli-Q water for five days, using a dialysis sack with MWCO of 3.5 kDa. Then, the dialyzed content was poured into Petri dishes and frozen at –20°C overnight, followed by lyophilization at 0.005 mbar and –90°C for 48 h, using a freeze-dryer Alpha 2-4 LSCbasic (Martin Christ GmbH, Steinheim, Germany).

Characterization methods:

Fourier Transform Infrared (FTIR) spectra were recorded on a FTIR spectrometer (Bruker Vertex 70, Billerica, MA, USA), equipped with an attenuated total reflectance (ATR) crystal, in absorbance mode in 4000–600 cm^{-1} wavenumber range, at 4 cm^{-1} resolution and 32 scans for each sample.

^{1}H-NMR spectra were registered on a NMR spectrometer (Bruker Avance III HD 600, Bruker Biospin, Rheinstetten, Germany) at a resonance frequency of 600.12 MHz. Prior to the analysis, the samples were solubilized in DMSO-d_6, or D_2O. Then, the samples were transferred to NMR tubes for analysis and the chemical shifts were displayed in parts per million (ppm) and the data were processed by the means of TopSpin 3.5 pl 6 software.

The binding efficiency of IMC to NH_2-PEG-NH_2 to form the pIMC, was estimated by UV-Vis spectrometry, by calculating the ratio between pIMC / IMC absorbance. The stock solutions were prepared in DMSO, and the IMC and pIMC samples were analyzed at concentrations of 0.05 mg/mL and 0.125 mg/mL, obtaining a binding efficiency around 19.23

± 1.5%, in agreement with other reported work [1]. The results of structural characterization were presented in **Figure S1 b-d.**

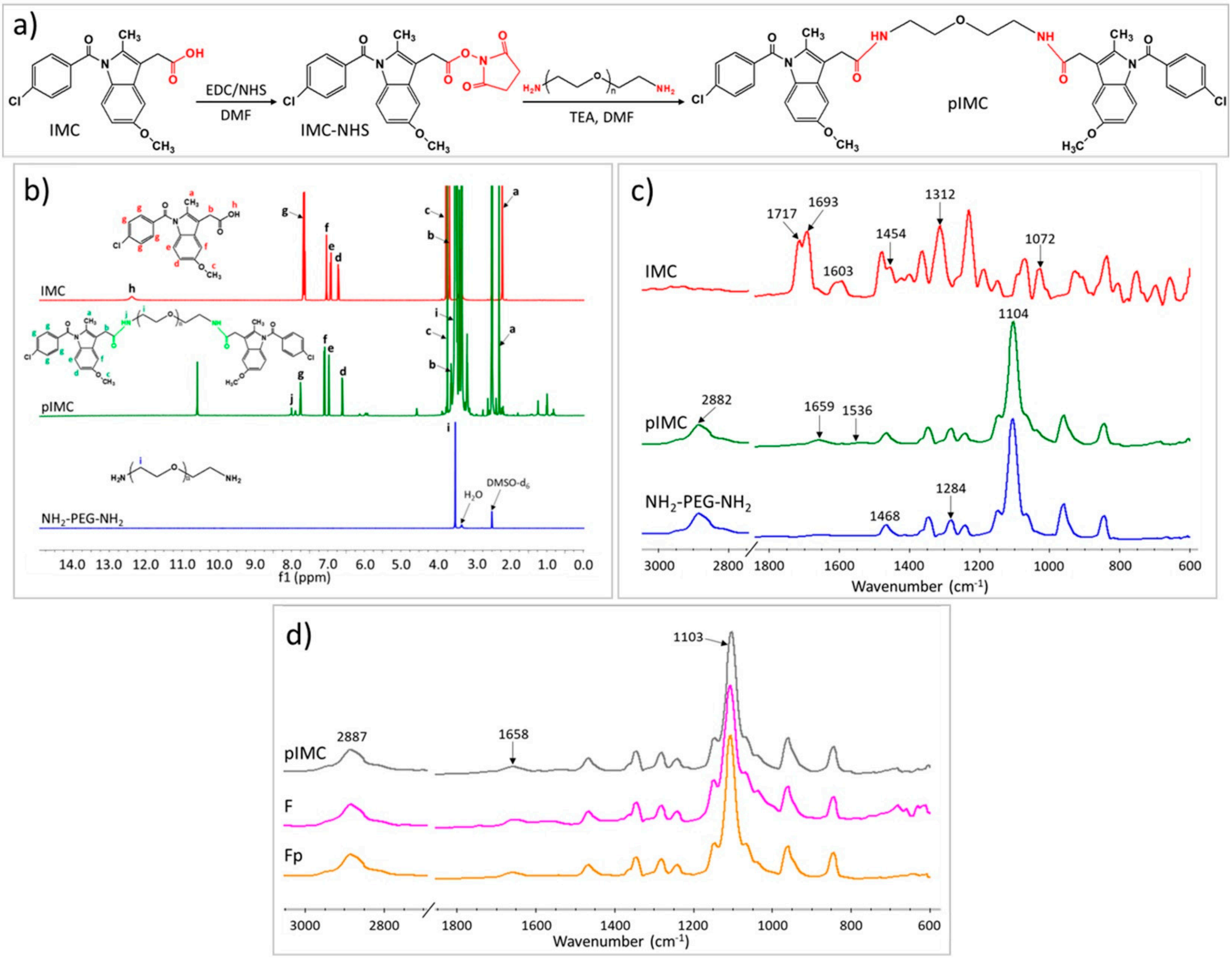

Figure S1. a) Obtaining reaction of pIMC by EDC/NHS coupling carbodiimide system; Structural characterization of pIMC: **b)** ^{1}H-NMR spectra of IMC, pIMC, NH_2-PEG-NH_2 and **c)** ATR-FTIR spectra of IMC, pIMC, NH_2-PEG-NH_2; **d)** ATR-FTIR spectra of pIMC, F, Fp.

The chemical structure of pIMC was evaluated by ^{1}H-NMR spectroscopy, thus confirming its successful synthesis through EDC/NHS coupling system-assisted reaction between -NH_2 groups of NH_2-PEG-NH_2 and -COOH groups of IMC. **Figure S1 b** were shown the ^{1}H-NMR spectra for IMC, NH_2-PEG-NH_2 and pIMC, in which the specific signals of DMSO-d_6 and the water from solvent were observed at 2.50 ppm and 3.33 ppm, respectively.

The ^{1}H-NMR spectrum of IMC was characterized by signals for methyl (-CH_3) group at 2.30 ppm (peak a), methylene (-CH_2-) group at 3.63 ppm (peak b), methoxy (O-CH_3) group at 3.72 ppm (peak c), structure of benzene nuclei at 6.60 ppm (peak d), 6.97 ppm (peak e), 7.09 ppm (peak f) and 7.75 ppm (peak g), and -COOH group at 12.38 ppm (peak h). These signals were also found in the ^{1}H-NMR spectrum of pIMC, except for the peak h corresponding to -COOH group which disappeared; the new peak (j) at 8.00 ppm was assigned to amide (-NH-

) group formed between -COOH group of IMC and $-NH_2$ group of NH_2-PEG-NH_2. Furthermore, the specific signal observed in the ^{1}H-NMR spectrum of NH_2-PEG-NH_2 was also highlighted in the pIMC spectrum at 3.51 ppm (peak i), being attributed to repeated ethylene glycol units from the NH_2-PEG-NH_2 structure [1].

Then, the synthesis of the pIMC was also confirmed by ATR-FTIR spectrometry, through which the new covalent bonds between IMC and NH_2-PEG-NH_2 were investigated (**Figure S1 c**). The characteristic absorption peaks for IMC are highlighted at 1717 cm^{-1} (ν C=O of -COOH), 1693 cm^{-1} (ν C=O of amide I), 1603 cm^{-1} (ν C=C of aromatic groups), 1454 cm^{-1} (δ O-CH_3 of methoxy group), 1312 cm^{-1} (ν C-O of phenyl groups), 1072 cm^{-1} (ν C-Cl), and 900 cm^{-1} – 600 cm^{-1} range can be attributed to C-H out of plane deformation from the aromatic rings [2,3].

The NH_2-PEG-NH_2 FTIR spectrum is characterized by the following peaks: 2886 cm^{-1} (ν C-H of methylene -CH_2-), 1468 cm^{-1} (ν C-N [4]), 1284 cm^{-1} (CH_2 twist), 1104 cm^{-1} (ν C-O-C of -O-CH_2-CH_2- units of PEG structure), and the region between 1000 – 800 cm^{-1} (ν CH_2-CO) [5].

The pIMC FTIR spectrum exhibited the typical peaks for NH_2-PEG-NH_2 at 2882 cm^{-1}, 1104 cm^{-1} and the domain between 1000 – 800 cm^{-1}, which proved its presence in the chemical structure of pIMC. This is also supported by the ^{1}H-NMR spectrum of pIMC in which the ethylene glycol units of the NH_2-PEG-NH_2 were highlighted by the presence of the 3.51 ppm signal (peak i). Furthermore, the absence of peak at 1717 cm^{-1} from the pIMC FTIR spectrum demonstrated the consumption of -COOH group from IMC structure, fact also proved by ^{1}H-NMR spectroscopy, through the disappearance of the 12.38 ppm signal (peak h) attributed to the -COOH group. At the same time, the formation of amide I (ν C=O) and amide II (N-H bending vibration) was confirmed by the appearance of new bands at 1659 cm^{-1} and 1536 cm^{-1} in FTIR spectrum of pIMC, which was correlated with the ^{1}H-NMR results and the appearance of a new signal at 8.00 ppm (peak j), attributed to the amide group.

Hereinafter, the synthetized pIMC was introduced within the nanofibrous scaffold structure and its presence was highlighted by FTIR spectrometry as shown in **Figure S1 d**. The FTIR spectrum of Fp presented the characteristic absorption bands of both pIMC and CS/PEO electrospun nanofibrous matrix.

2. *Synthesis and characterization of methacryloyl-modified gelatin (GM)*

GM was achieved by chemically grafting the methacrylamide groups of methacrylic anhydride (MA) on the gelatin (Gel) backbone [6], as shown in **Figure S2 a.** Briefly, a solution of 10% (w/v) Gel was prepared in PBS with *p*H = 8.45 at 50°C under magnetic stirring, until the entire amount of Gel was solubilized. Afterwards, 0.35 mL MA/g Gel was dropped to the Gel solution, under vigorous stirring, maintaining the *p*H around 7 – 8 with 5M NaOH solution. The mixture was kept at 50°C under magnetic stirring for 2 h, then it was purified against ultra-pure water using dialysis sacks with MWCO of 12 kDa, at 37°C for 96 h, frozen at –20°C overnight and lyophilized for 48 h, at 0.005 mbar and –90°C.

Characterization methods:

Structural characterization was performed by FTIR and ^{1}H-NMR spectrometry (**Figure S2 b-d)**. The methacrylation degree (MD) of GM, expressed as percent of amine groups modified with methacrylamide groups, was also determined by ^{1}H-NMR spectroscopy [7,8]. The MD of GM was calculated using relation (1).

$$\text{MD (\%)} = \left(1 - \frac{\text{Lysine integration signal of GM}}{\text{Lysine integration signal of Gel}}\right) \times 100 \quad (1)$$

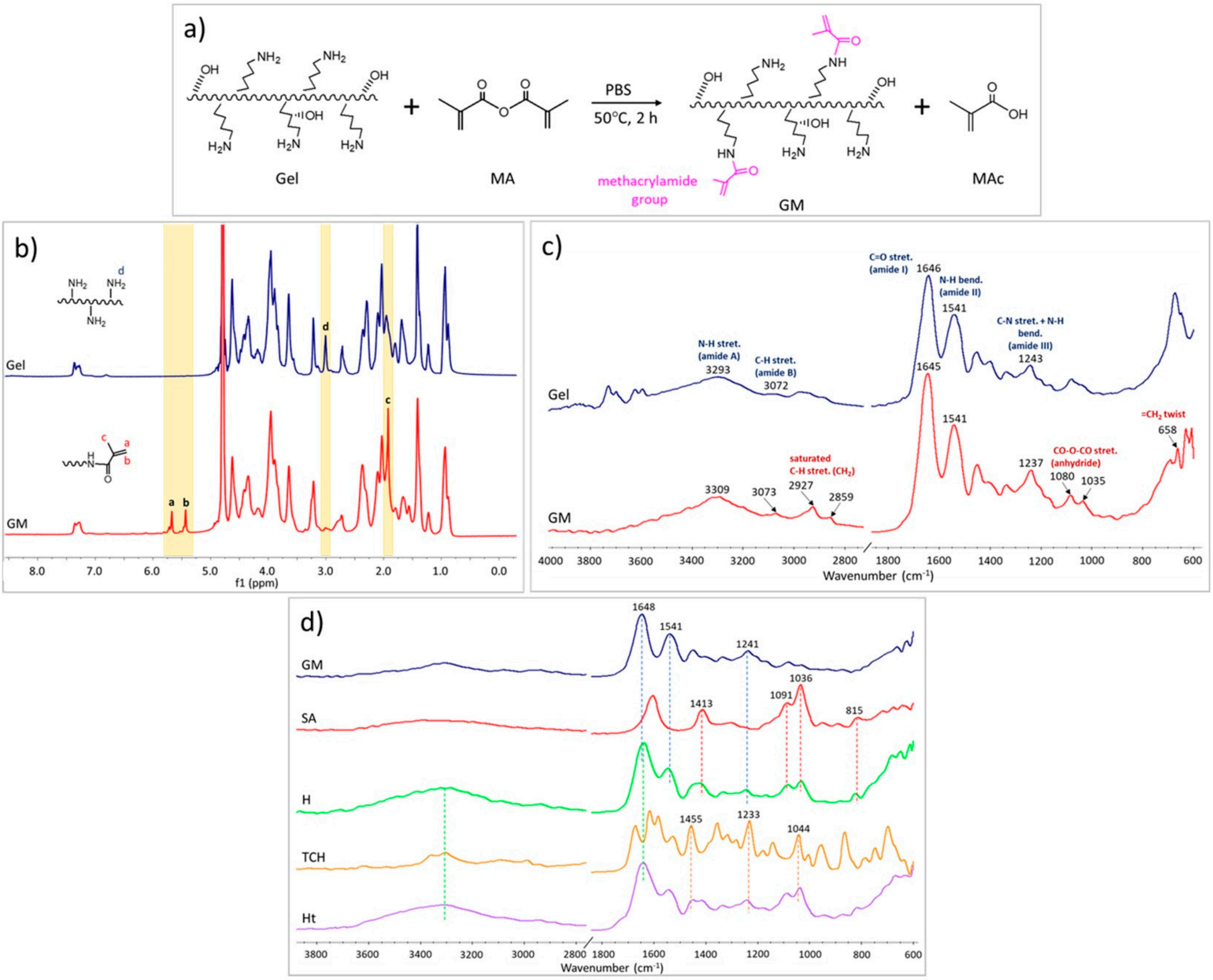

Figure S2. a) Obtaining reaction of GM. (Abbreviations: Gel – gelatin; MA – methacrylic anhydride; GM – methacryloyl-modified gelatin; MAc – methacrylic acid.) Structural characterization of GM: **b)** ^{1}H-NMR spectra of Gel and GM and **c)** ATR-FTIR spectra of Gel and GM; **d)** ATR-FTIR spectra of GM, SA, H, TCH, Ht.

The ^{1}H-NMR spectrum of GM showed new characteristic signals for methacryloyl groups, identified at 5.72 ppm–5.43 ppm which were attributed to the acrylic protons (2H) from vinyl group of MA (**Figure S2 b)**. Additionally, an increased in intensity of the signal at 1.92 ppm (methyl protons – 3H – of the grafted methacryloyl unit) and a decreased in intensity of the signal around 3.00 ppm (consumption of protons from lysine methylene – 2H [2]) in the GM spectrum compared to that of Gel, was be observed. These modifications proved the successful methacrylation of Gel, respectively the GM synthesis.

The spectra of Gel and GM were normalized to signals of phenylalanine aromatic ring around 7.35 ppm–7.27 ppm (5H), as an internal reference, because they were not involved in the methacrylation reaction. Then, the lysine integration signals of GM and Gel were used to

calculate the MD of GM [3]; MD is expressed as the percent of amine groups transformed to methacrylamide groups, and the obtained value was found to be around 76%.

Figure S2 c exhibited the FTIR spectra of pure Gel and synthesized GM. The Gel spectrum is characterized by the absorption peaks typical to protein secondary structure, at 3293 cm^{-1} (ν N-H of amide A), 3072 cm^{-1} (ν C-H of amide B), 1646 cm^{-1} (ν C=O of amide I), 1541 cm^{-1} (ν N-H of amide II) and 1243 cm^{-1} (ν C-N and N-H bending of amide III), which are also identified in the FTIR spectrum of GM. The presence of methacryloyl units in the chemical structure of GM was confirmed through the appearance of peaks at 2927 cm^{-1} and 2859 cm^{-1} (C-H symmetrical and asymmetrical stretching of aliphatic chains from methacrylate groups), 1080 cm^{-1} and 1035 cm^{-1} (ν C-O and ν CO–O-CO of MA), and 658 cm^{-1} (=CH_2 twisting from methacryloyl groups) [9].

Afterwards, a mixture of GM and SA solutions in a 1/1 ratio was prepared and used in 3D-printing of hydrogels (H), which were further loaded with TCH by physical adsorption (Ht). The chemical structures of 3D-printed H and Ht were also evaluated by FTIR spectrometry (**Figure S2 d)**.

FTIR spectrum of H showed both specific peaks of GM structure (1648 cm^{-1} - amide I, 1541 cm^{-1} - amide II, 1241 cm^{-1} - amide III) and peaks characteristic to SA structure (1413 cm^{-1} - symmetric ν COOH, 1091 cm^{-1} and 1036 cm^{-1} ν C–O–C and ν C–O, 815 cm^{-1} - mannuronic acid residues [10]). The shift of all these absorption peaks to higher values indicated the formation of non-covalent interactions between the two components of hydrogel. Furthermore, the specific peaks from TCH, e.g., 1455 cm^{-1} (skeleton vibration of the benzene ring in the TCH molecule [11]), 1233 cm^{-1} (ν C-N and ν C-C [12]) and 1044 cm^{-1} (ν C-OH), were also found in Ht FTIR spectrum, but their positions were slightly modified, probably owing to the hydrogen interactions with functionalities from hydrogel matrix.

References

1. Lin, J.; Pan, Z.; Song, L.; Zhang, Y.; Li, Y.; Hou, Z.; Lin, C. Design and in vitro evaluation of self-assembled indometacin prodrug nanoparticles for sustained/controlled release and reduced normal cell toxicity. *Appl. Surf. Sci.* **2017**, *425*, 674–681. [https://doi.org/10.1016/j.apsusc.2017.07.034]
2. Jain, D.K.; Darwhekar, G.; Solanki, S.S.; Sharma, R. Osmotically regulated asymmetric capsular system for sustained delivery of indomethacin. *J. Pharm. Investig.* **2013**, *43*, 27–35. [https://doi.org/10.1007/s40005-012-0050-3]
3. Ghitman, J.; Stan, R.; Ghebaur, A.; Cecoltan, S.; Vasile, E.; Iovu, H. Novel PEG-modified hybrid PLGA-vegetable oils nanostructured carriers for improving performances of indomethacin delivery. *Polymers* **2018**,*10*, 579. [https://doi.org/10.3390/polym10060579]
4. Resmerita, A.-M.; Assaf, K.I.; Lazar, A.I.; Nau, W.M.; Farcas, A. Polyrotaxanes based on PEG-amine with cucurbit [7] uril, *α*-cyclodextrin and its tris-O-methylated derivative. *Eur. Polym. J.* **2017**, *93*, 323-333. [https://doi.org/10.1016/j.eurpolymj.2017.06.015]
5. Vasi, A.-M.; Popa, M.I.; Tanase, E.C.; Butnaru, M.; Verestiuc, L. Poly (acrylic acid)–poly (ethylene glycol) nanoparticles designed for ophthalmic drug delivery. *J. Pharm. Sci.* **2014**, *103*, 676–686. [https://doi.org/10.1002/jps.23793]
6. Lee, J.; Park, C.H.; Kim, C.S. Microcylinder-laden gelatin-based bioink engineered for 3D bioprinting. *Mater. Lett.* **2018**, *233*, 24 – 27. [https://doi.org/10.1016/j.matlet.2018.08.138]
7. Ermis. M. Photo-crosslinked gelatin methacrylate hydrogels with mesenchymal stem cell and endothelial cell spheroids as soft tissue substitutes. *J. Mater. Res.* **2021**, *36*, 176-190. [https://doi.org/10.1557/jmr.2020.323]
8. Zu, G.; Meijer, M.; Mergel, O.; Zhang, H.; van Rijn, P. 3D-printable hierarchical nanogel-GelMA composite hydrogel system. *Polymers* **2021,** *13*, 2508. [https://doi.org/10.3390/polym13152508]
9. Mertz, G.; Fouquet, T.; Becker, C.; Ziarelli, F.; Ruch, D. A methacrylic anhydride difunctional precursor to produce a hydrolysis-sensitive coating by aerosol-assisted atmospheric plasma process. *Plasma Proc. Polym.* **2014,** *11(8),* 728-733. [https://doi.org/10.1002/ppap.201400050]

10. Belattmania, Z.; Kaidi, S.; El Atouani, S.; Katif, C.; Bentiss, F.; Jama, C.; Reani, A.; Sabour, B.; Vasconcelos, V. Isolation and FTIR-ATR and ^{1}H NMR characterization of alginates from the main alginophyte species of the Atlantic Coast of Morocco. *Molecules* **2020,** *25(18)*, 4335. [https://doi.org/10.3390/molecules25184335]
11. Chen, Z.; Mu, D.; Chen, F.; Tan, N. $NiFe_2O_4$@ nitrogen-doped carbon hollow spheres with highly efficient and recyclable adsorption of tetracycline. *RSC Adv.* **2019,** *9(19)*, 10445-10453. [https://doi.org/10.1039/C9RA00670B]
12. Ghazalian, M.; Afshar, S.; Rostami, A.; Rashedi, S.; Bahrami, S.H. Fabrication and characterization of chitosan-polycaprolactone core-shell nanofibers containing tetracycline hydrochloride. *Coll. Surf. A: Physicochem. Eng. Asp.* **2022,** *636*, 128163. [https://doi.org/10.1016/j.colsurfa.2021.12816]
